# Proportion of the Insane

**Published:** 1845-05

**Authors:** 


*Proportion of the Insane.*—Dr. Thurnam read a paper on the Statistics of Insanity, designed to refute Esquirol's theory that women were more predisposed to insanity than men in the proportion of 38 to 37. The discussion of this point turned chiefly on the corrections necessary to be made in statistical tables of insanity by taking into account the rates of mortality, the proportions of male and female population, and the probability of recovery in the two sexes. Dr. Thurman came to the conclusion that Esquirol's proportions ought to be reversed; but the question ultimately resolves itself into whether more confidence is due to the statistical returns of insanity in France or England,.

In the discussion that ensued, Mr. Tuke entered at length into an examination of the alleged disproportion of insane among the Society of Friends, tending to prove that the predisposition to mental alienation in that society is less than in the general community.

In the absence of Col. Sykes, Mr. Heywood read his paper on the Lunatic Asylums of Bengal. They are chiefly remarkable for the rapidity with which they have improved since their first establishment, and the very trifling cost at which they are supported.—*Meeting of the British Association for the Advancement of Science.*

